# Subwavelength imaging with a zero-mass sonic meta-atom

**DOI:** 10.1126/sciadv.adz9172

**Published:** 2026-03-04

**Authors:** Thibaut Devaux, Eun Bok, Jong J. Park, Sam H. Lee, Motonobu Tomoda, Osamu Matsuda, Oliver B. Wright

**Affiliations:** ^1^ Division of Applied Physics, Faculty of Engineering, Hokkaido University, Sapporo 060-8628, Japan.; ^2^ GREMAN UMR 7347, Université de Tours, INSA CVL, CNRS, 41000 Blois, France.; ^3^ Department of Physics and Engineering Physics, Yonsei University, Wonju 26493, Republic of Korea.; ^4^ Institute of Advanced Machines and Design, Seoul National University, 1 Gwanak-ro, Gwanak-gu, Seoul 08826, Republic of Korea.; ^5^ JJNS Co. Ltd., Daejeon 34002, Korea.; ^6^ Institute of Physics and Applied Physics, Yonsei University, Seoul 03722, Republic of Korea.; ^7^ Faculty of Science and Technology, Oita University, Oita City, Oita 870-1192, Japan.; ^7^ Hokkaido University, Sapporo 060-8628, Japan.; ^8^ Graduate School of Engineering, The University of Osaka, Yamadaoka 2-1, Suita, Osaka 565-0871, Japan.

## Abstract

Acoustic metamaterials offer powerful solutions for manipulating sound at subwavelength scales. One important application is super-resolved acoustic imaging, which relies on access to evanescent waves beyond the diffraction limit. Near-field techniques using subwavelength probes can capture these waves, revealing fine object details. Here, we introduce an experimental platform that harnesses airborne extraordinary transmission to couple evanescent acoustic waves into a subwavelength, zero-mass sonic meta-atom probe. By mounting a circular membrane at the tip of an air-filled waveguide with a conical tip, we exploit a modification of the acoustic inertance—caused by an object’s proximity—via the sonic Drexhage effect, leading to a downshift of the resonant frequency in the kilohertz range. Experimental results, supported by numerical and theoretical models, demonstrate that extreme subwavelength imaging is enabled by measuring the waveguide’s acoustic reflection coefficient, with lateral and depth resolutions of approximately λ/20 and λ/650, respectively (where λ is the acoustic wavelength). The platform’s capabilities for texture measurement and noncontact scanning are also demonstrated.

## Teaser

A zero-mass sonic meta-atom enables subwavelength imaging by sensing evanescent waves through extraordinary transmission.

## INTRODUCTION

Subwavelength optical and acoustic imaging using metamaterials has attracted much attention in recent years, relying on both negative effective parameters and superlensing concepts (*1*–*14*). In parallel, metamaterials and meta-atoms have been used to concentrate energy into regions much smaller than the wavelength through extraordinary transmission (ET) geometries based on subwavelength optical or acoustic resonators (*15*–*22*). In the acoustic domain, ET has been demonstrated for airborne sound, bulk acoustic waves in solids, and surface acoustic waves (*17*–*24*). Despite its potential to concentrate wave energy into tiny regions, the application of ET to subwavelength imaging remains unexplored. An acoustic device capable of capturing evanescent waves through ET could reveal fine details and subwavelength features of an object with minimal signal loss. Scanned probes of subwavelength size that convert airborne evanescent waves into propagating waves offer a promising route—one fundamentally distinct from air-based confocal acoustic microscopes (*25*, *26*). For example, the design of Molerón and Daraio (*27*)—based on tailoring the effective modulus—uses a square tube containing Helmholtz resonators scanned over an object to detect subwavelength object edges at kilohertz frequencies. However, the square cross section—which precludes lateral isotropy—and the limitation to edge detection both complicate image interpretation.

Here, we demonstrate that extraordinary acoustic transmission (EAT) in a zero-mass meta-atom probe—enabled by tailoring the effective mass rather than the modulus—provides subwavelength topographic imaging with airborne sound while maintaining lateral isotropy. Our approach extends the EAT concept proposed by Park *et al.* (*20*), in which a small resonant membrane is embedded in a rigid wall within an air-filled waveguide to achieve zero effective mass $M_{\text{eff}}\equiv \text{Re}(\Delta pS_{\text{mem}}/\ddot{\xi })$ of the membrane vibration, which corresponds to its on-resonance condition. Here, Δp is the differential pressure across the membrane of area $S_{\text{mem}}$ and ξ is the membrane displacement. In our design, the resonantly vibrating circular membrane is mounted at the end of a tube terminated with a conical tip. The proximity of an external object modifies the EAT by altering the acoustic inertance of the tip, thereby changing the acoustic reflectance inside the tube (*28*). The effective tip inertance, $I_{\text{eff}}\equiv \Delta p/\dot{Q}$, quantifies the pressure difference required to induce a unit change in the rate of volumetric flow Q. Unlike previous metamaterial-based imaging approaches, our method exploits the sonic Drexhage effect—originally observed in optics (*29*) and further elucidated in acoustics (*30*)—where the acoustic inertance of a vibrating disc is modified by the proximity of a reflective surface.

A related optical imaging technique, aperture-based near-field scanning optical microscopy (NSOM) (*31*–*33*), uses subwavelength apertures to achieve spatial resolution beyond the diffraction limit. In conventional NSOM, a metal-coated tapered optical fiber with a nanoscale aperture scans in close proximity to a sample, converting evanescent waves into propagating radiation that can be detected in the far field. Similarly, our EAT probe uses a subwavelength resonant membrane aperture to convert evanescent acoustic waves into propagating modes within a waveguide. A crucial difference, apart from the radiation involved, is that aperture-based NSOM converts evanescent optical fields through an aperture, whereas our EAT probe uses the resonant nature of a zero-mass meta-atom to achieve enhanced wave transmission through an aperture. This behavior is analogous to how surface plasmons can resonantly enhance optical transmission through subwavelength holes (*34*, *35*).

We show that our zero-mass meta-atom probe can image the topography of objects in two dimensions at kilohertz frequencies, achieving typical resolutions of ~λ/20 for the lateral direction and ~λ/650 for the depth direction, where λ is the acoustic wavelength. The probe’s circular symmetry ensures isotropic sensitivity in the lateral plane, yielding undistorted images without contact with the sample. We support our findings with analytical models and simulations, providing insights into the physics of the sonic Drexhage effect.

## RESULTS

### Demonstration of the zero-mass meta-atom probe operation

**Fig. 1.**
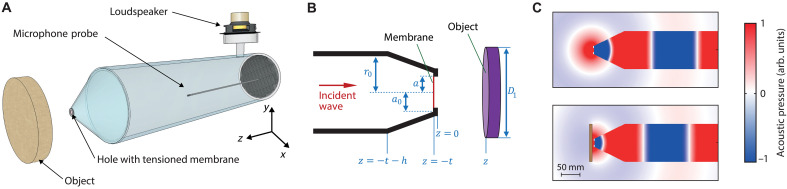
EAT-based acoustic probe geometry and simulated pressure fields. (**A**) Schematic diagram of the zero-mass meta-atom probe. The reflection coefficient of the tensioned membrane is measured inside the waveguide using a probe microphone. (**B**) Cross-sectional detail of the probe in the region of the conical tip. (**C**) Simulated cross-sectional map of the acoustic pressure inside and outside the probe for a rigid cylindrical object (diameter 100 mm, thickness 8 mm) placed at z=∞ (top image) and 4.2 mm (bottom image) on resonance at 1340 Hz, where the acoustic wavelength is ~23 times the membrane diameter.

A schematic of the zero-mass meta-atom probe is shown in Fig. 1A, and its cross-sectional detail is shown in Fig. 1B. It consists of a circular air-filled waveguide (internal diameter 2*r*_0_ = 100 mm, length 85 cm, wall thickness 5 mm), closed at one end by a 0.011-mm-thick polyethylene membrane of diameter *D* = 2*a* = 11.0 mm, tensioned and held in a rigid annular mount of thickness *t* = 1.5 mm. A stiff polyethylene truncated cone (axial length *h* = 80 mm, thickness 2 mm) is fitted between the waveguide and the annular mount, yielding a cone tip diameter of 2*a*_0_ = 22 mm. This tapered geometry allows access to samples of varied topography, reminiscent of a standard local probe configuration. A loudspeaker mounted off the opposite, anechoically terminated waveguide end provides tunable excitation. More experimental details are given in Materials and Methods.

**Fig. 2.**
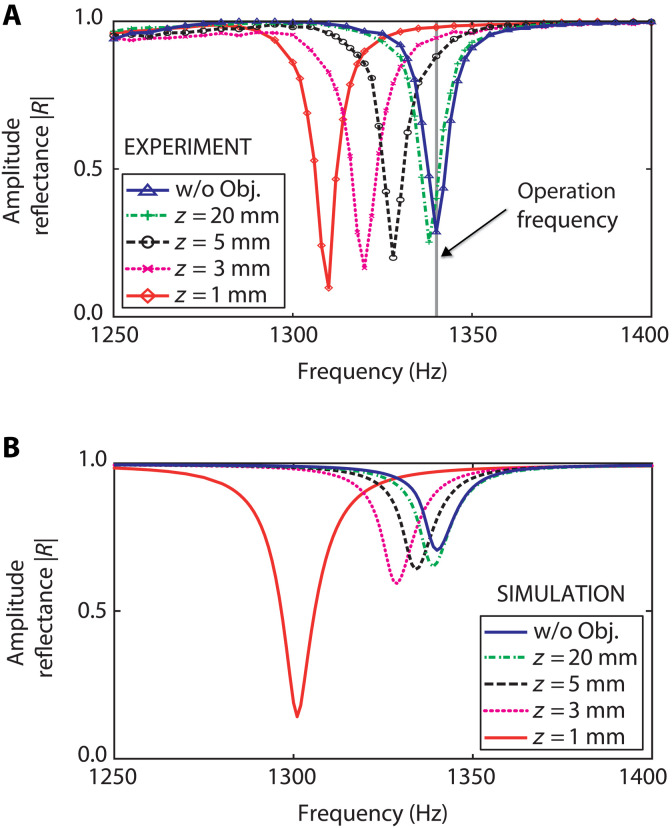
Experimental and simulated spectra of the EAT probe. Spectra of the modulus of the acoustic reflection coefficient ∣R∣ for different object positions in front of the probe. As the object approaches the membrane, both the reflection coefficient and the resonance frequency decrease. (**A**) Experimental results. (**B**) Numerical simulations obtained using FEM. The label “w/o Obj.” denotes the case without an object.

We first characterize the EAT of the zero-mass meta-atom by measuring the acoustic amplitude reflection coefficient ∣R∣ as a function of frequency f inside the waveguide in the absence of an external object, as shown in Fig. 2A (blue solid curve and triangles). At the membrane resonance $f_{0}=1340$ Hz, equivalent to an acoustic wavelength λ = 25.6 cm, we observe a minimum ∣R∣ with a resonance width corresponding to a *Q*-factor of 340.

On resonance, a substantial fraction of the incident acoustic energy is transmitted through the membrane for a filling factor of α=0.012 (i.e., 1.2%, the ratio of the hole area to the cylindrical waveguide cross-sectional area). Numerical simulations, described below, yield the following on-resonance results in the absence of an object: Γ=0.52 and τ=0.26, where $\Gamma ={|R|}^{2}$ is the energy reflection coefficient and τ is the energy transmission coefficient. These values correspond to an energy absorption coefficient A=1−Γ−τ=0.22. The coefficients are defined for the unit comprising the cone and the zero-mass meta-atom membrane. The unit’s input side corresponds to the cone-waveguide interface and its output side to free space. An effective ET efficiency for this unit can be defined in the standard way as η=τ/α=24, consistent with the system being in the regime of EAT (η>1) (*20*, *23*). (The experimentally determined value of |*R*| is somewhat smaller than in the simulation, suggesting that τ—and hence η—may be larger in the experiment.) In contrast, simulations of the identical system without the membrane at the same frequency (1340 Hz) yield Γ=0.9826 (≈0.98), τ=0.0064, A=0.011, and η=0.053. This demonstrates that the EAT membrane is essential for efficient device operation. On resonance, the membrane diameter corresponds to approximately λ/23.

A brief outline of the principle of operation of the EAT probe is as follows: With no object present, the on-resonance zero-mass meta-atom facilitates acoustic energy transmission, markedly lowering ∣R∣ compared to the case of a bare hole (*20*). An external object placed in front of the membrane induces wave reflection and a resonance frequency shift (*30*). When operating at a frequency fixed to the original resonance $f_{0}$, the net result is an increase in ∣R∣ inside the waveguide.

To characterize the sensing range, we measure ∣R(f)∣ for different axial object positions using a medium-density fiberboard (MDF) wooden disc of diameter *D*_1_ = 100 mm and a thickness of 8 mm, placed perpendicular to and centered on the z axis, as shown in Fig. 1A. (See Materials and Methods.) The acoustic amplitude reflection coefficient of this disc at normal incidence is predicted to be within 0.001 of unity. Figure 2A also shows the experimentally measured spectra ∣R(f)∣ for probe-object separations *z* = 1, 3, 5, and 20 mm. Equivalent spectra obtained by numerical simulation are shown in Fig. 2B (see Materials and Methods). The shift in resonance frequency and the decrease in the on-resonance value of ∣R∣ for lower values of z mirror the behavior observed experimentally. The difference between the simulated and experimental on-resonance values of ∣R∣ may arise from neglecting the structural damping of the membrane or the cone’s elastic rigidity in the simulations. (Shifting the position of the apparatus vertically by 30 cm did not affect the detected spectrum in the absence of an object, so it seems unlikely that reflections from the room walls were responsible.)

Figure 1C compares the simulated acoustic pressure field under two conditions (see Materials and Methods for simulation details). The top panel shows the field in the absence of an object, characterized by a spherically expanding wavefront. An approximate analytic expression for this extreme near-field pressure field, relevant to imaging resolution, is derived in Materials and Methods. The bottom panel illustrates the modification of this field due to the proximity of the object. The cross sections show that the object markedly distorts the wavefront from its original approximate spherical symmetry. Animations are provided in movie S1.

Before describing the imaging mode of the probe, we examine the physical origin of the image contrast. To this end, we present an analytical theory that relates the acoustic reflectance inside the tube to the sensed impedance and the acoustic inertance of the tip.

### Theory of probe operation

The operation of the probe can be modeled using a lumped element approach (*28*, *36*). The origin of the axial coordinate z is taken to be at the front of the annular housing, i.e., at the probe extremity. This analysis applies to any axially symmetric object placed outside the probe tip. The acoustic amplitude reflection coefficient for plane waves incident on the cone from inside the waveguide (see Fig. 1B) is given by

$$R=\frac{Z_{n}-Z_{c}}{Z_{n}+Z_{c}}$$ (1)

where $Z_{c}=\rho_{0}c_{0}/\pi r_{0}^{2}$ is the characteristic acoustic impedance of the tube divided by its cross-sectional area, and $Z_{n}$ is the corresponding impedance at the plane of the wide end of the cone. Here, $\rho_{0}$ and $c_{0}$ are the density and sound velocity of air, respectively.

As shown by the detailed theoretical calculation in the Supplementary Materials, the EAT is governed by the cone geometry and by the acoustic impedance $Z_{t}$ at the plane of the narrow end of the cone (*28*, *37*–*41*), where $Z_{t}$ includes a contribution from the membrane:

$$\begin{array}{cc} Z_{t}= & -i\omega \left[\frac{\rho_{0}\left(0.85a+t\right)}{\pi a^{2}}+\frac{m_{\mathrm{mem}}}{{\left(\pi a^{2}\right)}^{2}}\right]\\ & -\frac{8\pi \tau }{i\omega {\left(\pi a^{2}\right)}^{2}}+\frac{b_{h}}{{\left(\pi a^{2}\right)}^{2}}+Z_{0} \end{array}$$ (2)

where $m_{\text{mem}}$ is the membrane mass, τ is the membrane pre-tension (unrelated here to the energy transmission coefficient defined earlier), ω is the angular frequency, $b_{h}$ is a dissipative damping term associated with the hole, and $Z_{0}$ is the acoustic impedance at the plane *z* = 0, i.e., at the probe extremity. Clearly, the experimentally measured ∣R∣ varies with the object-dependent impedance $Z_{0}$.

The effective acoustic inertance is related to $Z_{t}$ through $I_{\text{eff}}=Z_{t}/\left(-\mathrm{i\omega}\right)$ and to the effective membrane mass $M_{\text{eff}}$ through $I_{\text{eff}}=M_{\text{eff}}/{\left(\pi a^{2}\right)}^{2}+{\mathrm{ib}}_{\text{eff}}/\left[\omega {\left(\pi a^{2}\right)}^{2}\right]$, where $b_{\text{eff}}$ is an effective damping coefficient that includes the radiation contribution associated with coupling to free space (i.e., the free-space radiation impedance). In (*20*, *23*), by contrast, $b_{\text{eff}}$ accounts only for energy dissipation. The effective mass is, in turn, related to the Rayleigh end correction of the hole (*23*, *28*). To understand how variations in inertive loading affect ∣R∣, a relation between the impedances $Z_{n}$ and $Z_{t}$ is required; this relation is derived analytically in the detailed theory (see the Supplementary Materials).

In the absence of an object, ∣R∣ is primarily governed by the impedance mismatch at the transition between the membrane tube and free space.

When the probe extremity approaches an object, the real part of the effective acoustic inertance $I_{\text{eff}}$ increases, leading to an increase in ∣R∣. At the same time, the resonance shifts to lower frequencies, as expected from the increased inertive loading owing to the more confined geometry outside the probe tip. This behavior can be explained by modeling the object in front of the membrane as an in-phase image monopole. The resulting back-action modifies the radiation impedance $Z_{0}$—both its real and imaginary parts—as described in (*30*). This is the sonic Drexhage effect, which gives rise to the resonance frequency downshift.

### Axial scanning mode

**Fig. 3.**
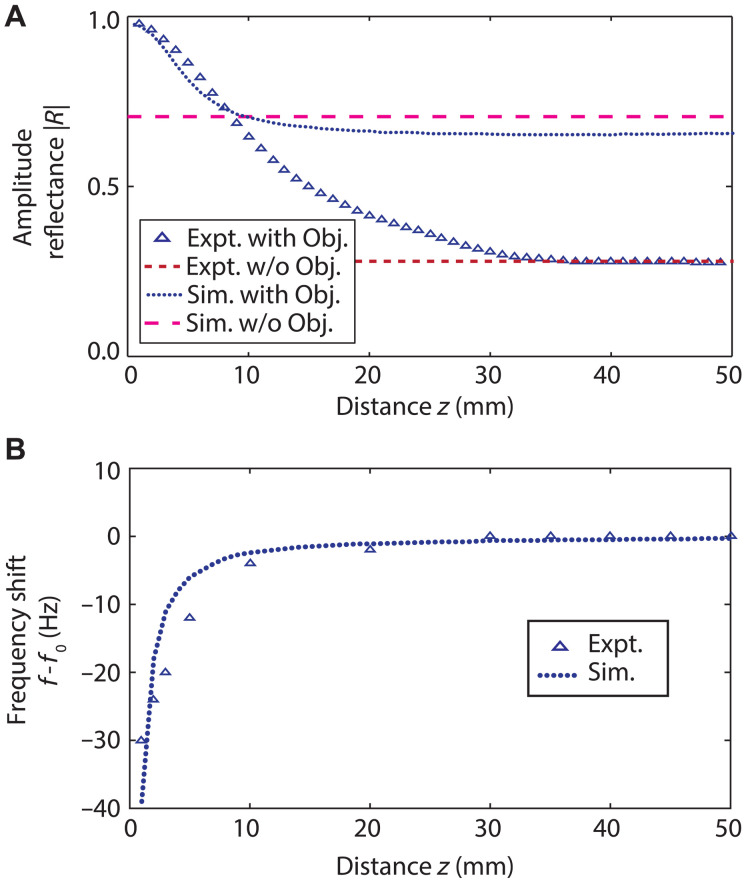
EAT probe response as a function of the object distance. (**A**) Modulus of the acoustic reflection coefficient |R| as a function of the object distance z measured at the membrane resonance frequency $f_{0}=1340$ Hz, which is determined with no object present. Results in the absence of an object are shown by the dashed line. The legend labels “w/o Obj.”, “Expt.”, and “Sim.” denote the cases without an object, experiment, and simulation, respectively. (**B**) Resonance frequency shift as a function of object distance.

To characterize the sensing range, we measure the modulus of the acoustic reflection coefficient ∣R∣ inside the tube at the membrane resonance frequency $f_{0}$ as a function of the object axial distance *z*, as shown in Fig. 3A. This mode of operation corresponds to the zero-mass condition (i.e., $M_{\text{eff}}=0$) or, equivalently, to $I_{\text{eff}}={\mathrm{ib}}_{\text{eff}}/\left[\omega {\left({\text{π}a}^{2}\right)}^{2}\right]$, which holds before the introduction of the object. Over the range z<30 mm, the object distance strongly influences the acoustic reflection coefficient, as expected because of the modified external geometry. We find that ∣R∣ decreases quasiexponentially as z increases, from a maximum value of 0.95 at *z* = 1 mm. For z>30 mm, the object position has a negligible influence on ∣R∣, which approaches the value 0.28 obtained on resonance in the absence of an object (see Fig. 2A). A suitable calibration of these results therefore allows the object distance to be determined. The depth of field is limited to ~3 membrane diameters, consistent with the extreme near-field nature of the probing.

Figure 3A also shows the results of simulations, which align with the general trends observed in the experiment. Oscillations in the reflection coefficient as a function of z are a hallmark of the sonic Drexhage effect and have also been noted in analogous experiments on acoustic gongs placed near a wall (*30*). These oscillations decrease in magnitude as the object diameter is reduced. For the chosen diameter, they are essentially damped out and predicted to be very small. Although simulations indicate that they begin to appear for *z* > 30 mm—as a barely visible change in slope in the dotted line in Fig. 3A—our experimental resolution is insufficient to reproduce them. The simulations without an object show a higher value of ∣R∣ at large *z* compared to the corresponding experimental value, a discrepancy that was identified and explained in connection with Fig. 2B.

Another possible mode of probe operation is to track the resonance frequency shift. Figure 3B shows this shift, $f-f_{0}$, as a function of the axial distance, z, derived from the data in Fig. 2. A pronounced dependence of $f-f_{0}$ on z is observed at short distances (≲10 mm) in both experiment and simulation, showing reasonable agreement. In both cases, the frequency approaches a constant value for z>30 mm, consistent with the absence of an object.

### Subwavelength imaging

An acoustic imaging system for topography can be realized through lateral (*x*-*y*) scanning of the probe. To demonstrate this capability, we first perform one-dimensional (1D) scanning using an MDF wooden plate with dimensions of 40 mm (width), 126 mm (length), and 8 mm (thickness), placed on a 100 mm–by–100 mm–by–3 mm brass plate. The scan is performed along the *x* axis at the center of the plate width, corresponding to approximately λ/6 at resonance.

**Fig. 4.**
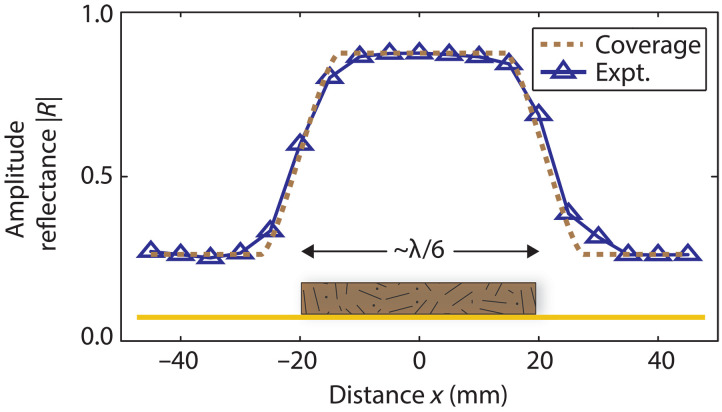
Experimental results for 1D lateral scanning. Modulus of the acoustic reflection coefficient (∣R∣) inside the tube versus distance along the x axis for an MDF wooden plate with a width of 40 mm (≈λ/6) and a thickness of 8 mm. Dashed lines represent the calculated ∣R(x)∣ based on the effective coverage ratio of the probe by the object at the distance z=2 mm, corresponding to an effective membrane diameter of 13 mm.

Figure 4 shows the modulus of the reflection coefficient, ∣R∣, measured along the *x*-axis at a constant frequency $f_{0}=1340$ Hz, with the probe-object distance fixed at z=2 mm. This small value of z is chosen to maximize lateral resolution while minimizing collision risk. The dashed lines in Fig. 4 represent the calculated profile ∣R(x)∣, derived from the effective coverage ratio (F) of the probe by the object at the chosen z (see Materials and Methods). The full width at half maximum (FWHM) of dF/dx at the object edges defines a lateral resolution of approximately 13 mm, i.e., ~λ/20. The agreement between the calculated and measured ∣R(x)∣ profiles is excellent . The small difference in the absolute value of |*R*| at *z* = 2 mm relative to that in Fig. 3A is attributed to differences in the object’s lateral dimensions and minor temperature variations. This lateral resolution, obtained under extreme near-field conditions, depends on both the membrane diameter and the probe-object distance z (see Materials and Methods).

The depth resolution must also be evaluated. On the basis of the experimental noise floor, the estimated reflectance resolution is Δ∣R∣≈0.01. At z=2 mm, where $|d|R|/\mathrm{dz}|\approx 0.025\;{\text{mm}}^{-1}$, the corresponding depth (out-of-plane) resolution is Δz≈0.4 mm (~λ/650).

Despite its subwavelength size, the object is well resolved.

**Fig. 5.**
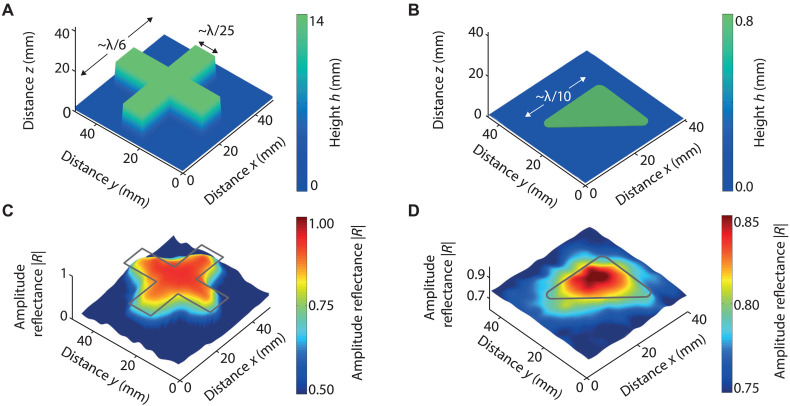
2D topography imaging. (**A** and **B**) Object geometries: an MDF wooden cross (thickness 14 mm) and a polyurethane rubber triangle (thickness 0.8 mm). (**C** and **D**) Measured modulus of the acoustic reflection coefficient (∣R∣) as a function of x and y. Gray lines indicate the object positions.

We next turn to 2D imaging of structured objects. First, we scan an MDF wooden cross with an arm width of 10 mm, which corresponds to ~λ/25 at our operating frequency ($f_{0}$), a thickness of 14 mm (~λ/18), and an overall width of 40 mm (~λ/6), as shown by the geometry of Fig. 5A. The cross is placed on the brass plate described above, and the imaging distance (to the top surface) is again set to *z* = 2 mm. Under identical conditions, we also image a right-angled black polyurethane rubber triangle with rounded vertices, as shown in Fig. 5B. (See Materials and Methods for further details.) The triangle has a side length of 27 mm before rounding (~λ/10), a height of 0.8 mm (~λ/320), and a vertex radius of curvature of 3 mm. (See Materials and Methods for further details.)

Figure 5 (C and D) show the corresponding 2D amplitude reflectance (∣R∣) images. In both cases, the objects are clearly resolved. For the wooden cross, the EAT probe resolves geometric features with a lateral precision of ~λ/20 (~13 mm), consistent with the 1D scanning experiment. The edge-profiling accuracy is limited by the effective partial coverage. A similar lateral resolution is obtained with the polyurethane triangle. The depth resolution in both cases is ~λ/650 (~0.4 mm).

These experimental results show that the EAT probe can image the topography of materials, including relatively soft ones, in two dimensions with deep subwavelength resolution. The object’s acoustic reflection coefficient strongly influences the probe response when its acoustic impedance is comparable to that of air, as in the case of foams (see the Supplementary Materials for an experimental illustration) (*42*, *43*).

## DISCUSSION

We harness the sonic Drexhage effect for near-field acoustic sensing using EAT in a tensioned membrane meta-atom. By measuring the acoustic reflection coefficient ∣R∣ in a waveguide, we detect object proximity through changes in ∣R∣ arising from a downshift of the membrane resonance frequency. This frequency shift results from perturbation of the effective acoustic inertance at the probe tip, enabling deeply subwavelength resolution in both lateral and depth directions through evanescent coupling.

We demonstrate 2D imaging in constant-frequency mode that captures the topography of wood and polyurethane structures, revealing their subwavelength features. This noncontact method is applicable to a wide range of materials. In the near-field, we achieve an isotropic lateral resolution of ~λ/20, surpassing the diffraction limit of far-field focusing devices. The out-of-plane resolution of ~λ/650 promises precise mapping of minute topographic variations.

Incorporating a two-microphone method would extend the EAT probe to measurement of the complex reflection coefficient R, enhancing sensitivity to sample elasticity and density. However, in this study, we concentrate on acoustically hard objects with near-unity reflection coefficients. Moreover, scaling down the probe dimensions for ultrasonic operation could broaden the range of EAT applications by improving spatial resolution—enabling, for example, higher-resolution lensless imaging of fabrics or human skin at frequencies far below those required by conventional acoustic microscopes. Finally, implementing a feedback loop to maintain resonance during imaging would yield resonance-frequency images, providing complementary insights into sample properties.

## MATERIALS AND METHODS

### Experimental setup

The cylindrical acrylic waveguide, with a wall thickness of 5 mm, is terminated at one end by an anechoic wall. At the other end, the zero-mass meta-atom is created using a low-density (700 kg m^−3^) polyethylene membrane tensioned and fixed to the inner side of a 1.5-mm-thick metal washer (the annular mount). A small epoxy dot (mass 2.6 mg, diameter 2.5 mm) was added at the membrane center to increase the modal inertia and thereby reduce the relative damping of the resonance. The washer is attached to the thin end of the plastic cone (half-angle 26°), whose thick end is seamlessly connected to the acrylic waveguide.

A loudspeaker located at a distance of ~160 mm from the anechoic end of the waveguide provides the audio excitation. We use a microphone probe (Neosonic NS-9600) with lock-in amplifier detection, combined with the standing wave ratio (SWR) method to determine the modulus of the acoustic reflection coefficient in the waveguide at room temperature.

The 2D *x*-*y* scanning interval is 0.3 mm. The 2D scanning results in Fig. 5 are linearly interpolated for ease of viewing.

The density of the MDF is measured to be 785 kg m^−3^, and its longitudinal sound velocity is approximately 2100 m s^−1^ (*44*). The polyurethane rubber triangle is fabricated from a sheet (Bumpon SJ5832, 3M). Its density is measured to be 1500 kg m^−3^, and its longitudinal sound velocity is estimated as 1300 m s^−1^ (*45*). Both have acoustic reflection coefficients in air very close to unity.

### Coverage ratio and lateral resolution

The key parameter affecting the 1D scanning results (of Fig. 4) is the coverage ratio, defined here as the fraction of the membrane aperture area lying on one side of a straight-edge plane, expressed as

$$F\left(x\right)=\frac{a^{2}{\text{cos}}^{-1}\left(x/a\right)-x\sqrt{a^{2}-x^{2}}}{\pi a^{2}}$$ (3)

where x is the lateral offset of the membrane center from the straight interface defining the object boundary, and a is the membrane radius. For x=a, the interface lies at the probe edge and no coverage occurs, so F(a)=0. For, x=−a the probe is fully covered, giving F(−a)=1. To compare with measured data, the geometric coverage ratio is scaled to the magnitude range of the reflection coefficient according to

$$r\left(x\right)=\left(|R_{2}|-|R_{1}|\right)F\left(x\right)+\left|R_{1}\right|$$ (4)

where $|R_{1}|$ and $|R_{2}|$ denote the reflection coefficient magnitudes when the probe is fully over medium 1 and medium 2, respectively. For air, $|R_{1}|$ corresponds to ∣R∣ in the absence of an object, as determined from the data of Fig. 3A.

The lateral sensitivity of the probe is quantified by the spatial derivative of the coverage fraction, given by

$$\frac{\mathrm{dF}}{\mathrm{dx}}=-\frac{2}{\pi a^{2}}\sqrt{a^{2}-x^{2}},\;-a\leq x\leq a$$ (5)

attaining its maximum magnitude when the interface passes through the membrane center (at x=0), where ${|\mathrm{dF}/\mathrm{dx}|}_{\text{max}}=2/\left(\pi a\right)$. A practical measure of lateral resolution can be defined by the FWHM of ∣dF/dx∣, corresponding to the distance between the points where $|\mathrm{dF}/\mathrm{dx}|={|\mathrm{dF}/\mathrm{dx}|}_{\text{max}}/2$, yielding $x=\pm \left(\sqrt{3}/2\right)\;a$. The resulting FWHM is thus $\Delta x_{\text{FWHM}}\left(0\right)=\sqrt{3}\;a\approx 0.87\;D$, where D=2a. This provides a geometry-based estimate of the minimal resolvable separation between two interfaces under ideal conditions (i.e., *z* = 0).

The ideal geometric resolution estimate above assumes the object is in direct contact with the aperture (z=0). In the extreme near-field regime relevant to our experiment (*z* ≪ λ), the resolution is primarily determined by geometry rather than diffraction. A rapid degradation of resolution with increasing z is therefore expected, arising from the spherical spreading of waves emanating from a subwavelength aperture (see Fig. 1C). When λ ≫ *D*, as in our case (λ/D≈23), the acoustic pressure field spreads approximately isotropically. A rough estimate suggests that, for *z* ≪ *D*, the FWHM beam width increases as $\Delta x_{\text{FWHM}}\left(z\right)\approx \Delta x_{\text{FWHM}}\left(0\right)+\kappa z$, where κ is a constant representing natural spherical expansion rather than diffraction-limited broadening.

To estimate κ, consider a circular membrane of radius a clamped at the edge. The fundamental vibrational mode has an out-of-plane displacement amplitude given by (*46*)

$$w\left(r\right)=w_{0}J_{0}\left(\frac{\alpha_{01}r}{a}\right),\;\alpha_{01}\approx 2.4048$$

where r is the radial coordinate and $w_{0}$ the maximum center amplitude. The corresponding normal velocity is $v\left(r\right)=i\omega_{0}w\left(r\right)$, where ω_0_ is the resonance angular frequency.

The radiated pressure in the forward direction is obtained from the Rayleigh integral, which is strictly valid for a membrane mounted in an infinite rigid baffle—an approximation justified in the extreme near field owing to the presence of the washer (*28*):

$$p\left(\rho ,z\right)=\left|i\rho_{0}\mathrm{ck}\iint_{S}v\left(r^{\prime }\right)\frac{e^{\mathrm{ik}|r-s|}}{2\pi |r-s|}\;\mathrm{dS}\right|$$

where $\mathrm{dS}=r^{\prime }\;dr^{\prime }\;d\phi$, with $\left(r^{\prime },\phi \right)$ denoting polar coordinates on the membrane surface (S). The vectors $s=\left(r^{\prime }\text{cos}\phi ,r^{\prime }\text{sin}\phi ,0\right)$ and r=(ρ,0,z) denote positions on the membrane and in the observation plane, respectively. Here, ρ is the radial coordinate in the observation plane, z is the axial distance from the membrane, ρ_0_ is the air density, c is the sound speed, and k is the wave number. (For simplicity, we treat the membrane as flush with the washer front, although in the experiment, it is recessed.) Pressure p refers to the amplitude of the time-harmonic acoustic field, i.e., the modulus of the oscillatory pressure. Cylindrical symmetry allows reduction of the Rayleigh integral to a single radial integral, with an inner integral over the azimuthal angle:

$$p\left(\rho ,z\right)=\left|i\rho_{0}\mathrm{ck}\int_{0}^{a}v\left(r^{\prime }\right)\;r^{\prime }\left[\int_{0}^{2\pi }\frac{\text{exp}\left(\mathrm{ik}\sqrt{\rho^{2}+{r^{\prime }}^{2}-2\rho r^{\prime }\text{cos}\phi +z^{2}}\right)}{2\pi \;\sqrt{\rho^{2}+{r^{\prime }}^{2}-2\rho r^{\prime }\text{cos}\phi +z^{2}}}\;d\phi \right]dr^{\prime }\right|$$

Numerical evaluation of this integral for D=11 mm (a=5.5 mm) and f=1340 Hz (λ≈0.256 m), using the fundamental-mode membrane velocity profile, confirms that the pressure decays rapidly with *z*. For example, p(0,2 mm)/p(0,0)=0.59 and p(0,10 mm)/p(0,0)=0.19. The lateral FWHM of the pressure magnitude also increases with distance. Numerical evaluation of the integral at z=0, 2, and 10 mm yields $\Delta x_{\text{FWHM}}^{\left(p\right)}\approx 9.4$, 13.0 and 36.5 mm, respectively. At z=2 mm, the effective lateral spreading parameter κ in the geometric parametrization $D_{\text{eff}}=0.87D+\kappa z$ is obtained by setting $D_{\text{eff}}$ equal to the calculated $\Delta x_{\text{FWHM}}^{\left(p\right)}$, yielding κ≈1.8. This geometric approach, based on the coverage ratio, provides an excellent fit to the lateral scanning data at z=2 mm shown in Fig. 4. At z=10 mm, κ≈2.7. The maximum lateral resolution is obtained at z=0, where $\Delta x_{\text{FWHM}}^{\left(p\right)}\approx 9.4\;\text{mm}\approx \lambda /30$.

Alternatively, from this value of FWHM at z=0, one can define a modified parameter κ′ such that $D_{\text{eff}}\left(z\right)=\Delta x_{\text{FWHM}}^{\left(p\right)}\left(0\right)+\kappa^{\prime }z$. Using the numerical results, we obtain values of κ′ at z=2 and 10 mm that are comparable to κ, yielding a consistent parametrization of the lateral spreading. (The value $\kappa^{\prime }\approx 1.6$ applies for z≤1 mm.) Although the full-wave FWHM of ∣p∣ provides a physically accurate measure of the lateral resolution, the coverage ratio approach offers a simpler, analytically based geometric estimate of the fraction of the object probed. It therefore provides an intuitive framework for interpreting 1D scanning data at small *z*.

### Numerical simulations

Numerical simulations are performed using the finite element method (FEM) with the commercial software COMSOL Multiphysics (version 5.3), assuming axial symmetry. In addition to the parameters given above, the following conditions were used: ambient temperature: 20°C; membrane tension: 49.5 N m^−1^; plane wave acoustic source with 0.02-Pa pressure amplitude, diameter of the free space around the device: 1000 mm; atmospheric pressure: 1 atm. Apart from the membrane, all solids are assumed to be perfectly rigid, and the bulk viscosity of air is neglected. Air properties were taken as density $\rho_{0}=1.2$ kg m^−3^, sound velocity $c_{0}=343$ m s^−1^, dynamic shear viscosity $\mu =1.813\times 10^{-5}$ Pa s, thermal conductivity κ=0.0258 W m^−1^ K^−1^, specific heat capacity $C_{p}=1005.4$ J kg^−1^ K^−1^, and ratio of specific heats γ=1.400 (*47*). The “Plane Wave Radiation” condition was applied at the boundary at the end of the tube to prevent reflections.

The FEM mesh consists of ~17,000 domain elements and 900 boundary elements. The maximum and minimum sizes of the domain elements are 55 and 0.05 mm, whereas those for the boundary and edge elements are 0.1 and 0.01 mm.

The FEM prediction accuracy, excluding systematic errors arising from the chosen physical constants, is estimated to be ~4%.
