# Supplementary material for: Subwavelength imaging with a zero-mass sonic meta-atom
Source: Sci Adv. 2026 Mar 4;12(10):eadz9172. doi: 10.1126/sciadv.adz9172 (PMC12959413; doi:10.1126/sciadv.adz9172)
Supplement: Supplementary file 1 — Supplementary Text Figs. S1 to S3 Legend for movie S1 [file sciadv.adz9172_sm.pdf]

Science Advances
AAAS

Supplementary Materials for

Subwavelength imaging with a zero-mass sonic meta-atom

Thibaut Devaux *et al.*

Corresponding author: Oliver B. Wright, olly@eng.hokudai.ac.jp

Sci. Adv. **12**, eadz9172 (2026)
DOI: 10.1126/sciadv.adz9172

The PDF file includes:

Supplementary Text
Figs. S1 to S3
Legend for movie S1

Other Supplementary Material for this manuscript includes the following:

Movie S1

1 Acoustic impedance relations for the conical-tip probe

1. Derivation of the impedance Z_n

The detailed geometry of the tip of the Extraordinary Acoustic Transmission (EAT) probe used in the analytical theory and simulations is shown in Fig. S1. As described in the main text, the principle is based on the reflection at a sub-wavelength membrane-covered hole, which depends on the distance to the object. This section explores the relationship between the impedance before and after the conical section of the probe.

In Fig. S1, the incident acoustic wave travels from left to right. The reflection coefficient, R, is defined as the ratio of the complex amplitude of the reflected wave to that of the incident wave at the interface $z = -t - h$, representing the boundary between the main tube and the conical horn. (The power reflection coefficient in the main tube region, where $z < -t - h$, is given by $|R|^2$). Contrary to the experimental situation, we assume that the membrane is placed at the center of its containing tube, with negligible effect on the calculations.

The coefficient R is expressed as

$$R = \frac{Z_n - Z_c}{Z_n + Z_c} \tag{S1}$$

where Z_n is the acoustic impedance at the plane $z = -t - h$, and $Z_c = \rho_0 c_0/\pi r_0^2$ is the characteristic acoustic impedance of air divided by the tube cross-sectional area in the region $z < -t - h$ (36). Here, r_0 is the tube radius, ρ_0 is the mass density of air, and c_0 is the sound velocity of air .

The acoustic impedance Z_n is defined as the average pressure field $\bar{p}$ divided by the volume velocity q at the interface. Z_n depends on the geometrical and mechanical conditions in the region where $z > -t - h$. To calculate the reflection coefficient, we first derive an expression for the impedance Z_n:

$$Z_n \equiv \left.\frac{\bar{p}}{q}\right|_{z=-t-h} = \left.\frac{\int_S p dS/\pi r_0^2}{\int_S \mathbf{u}\cdot\hat{\mathbf{z}} dS}\right|_{z=-t-h}, \tag{S2}$$

$$\bar{p}(z) \equiv \frac{1}{\pi r_0^2}\int_S p\, dS, \qquad q(z) \equiv \int_S \mathbf{u}\cdot\hat{\mathbf{z}}\, dS, \tag{S3}$$

where S represents the plane perpendicular to the z-axis in the horn domain ($-t - h \leq z \leq -t$). The pressure p and particle velocity $\mathbf{u}$ correspond to the waves propagating in the conical horn, shown in Fig. S2. We shall assume that the acoustic field inside the conical section can be approximately described as the lowest-order (axisymmetric) solution about the virtual apex O' (Fig. S2), i.e. as spherically spreading waves, under the condition $r_0 < \lambda/2$,

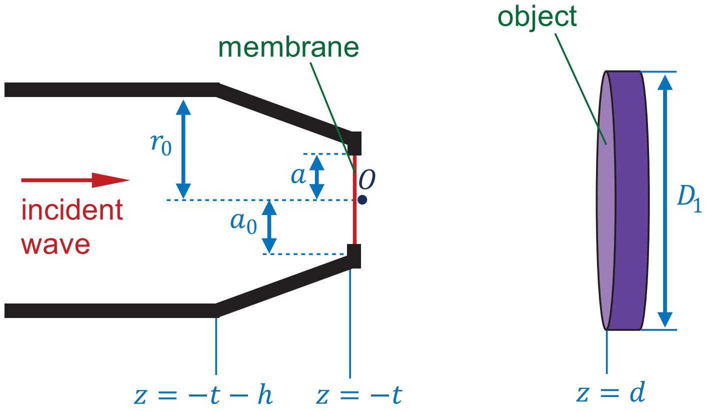

Fig. S1: Schematic of the EAT-based probe.

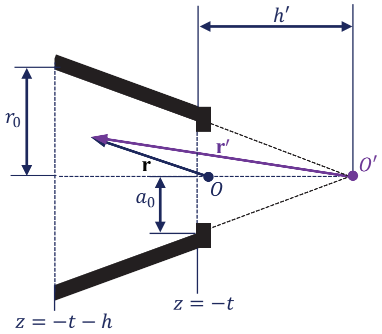

Fig. S2: Schematic of the conical horn part of the EAT-based probe. The primed coordinate system has origin O' situated at a point corresponding to the vertex of the truncated conical horn.

where λ is the wavelength, without further discussion of horn theory (28, 37, 38, 39, 40). This approximation is appropriate when higher-order angular modes are negligible (i.e., for an acoustically small cross-section). The validity of this assumption is later confirmed by comparison of the analytical results with Finite Element Method (FEM) simulations. The fields are given by

$$p(r') = p_+ \left(\frac{e^{-ikr'}}{r'} + \Gamma \frac{e^{ikr'}}{r'} \right). \tag{S4}$$

$$\mathbf{u}(r') = -\hat{\mathbf{r}'}\frac{p_+}{\rho_0 c_0}\left(\left[1+\frac{1}{ikr'}\right]\frac{e^{-ikr'}}{r'} - \Gamma\left[1-\frac{1}{ikr'}\right]\frac{e^{ikr'}}{r'}\right), \tag{S5}$$

which are expressed using the prime coordinate system ($\mathbf{r}' \equiv \mathbf{r} - (\mathbf{h}' - \mathbf{t})\hat{\mathbf{z}}$ and $\hat{\mathbf{r}'} \equiv \mathbf{r}'/r'$), as shown in Fig. S2, and terms $\exp(-i\omega t)$ have been omitted. The wavenumber is given by $k=\omega/c_0$, assuming lossless propagation. The term proportional to $(p_+/r')e^{-ikr'}$ represents the wave propagating toward the aperture (decreasing r' in Fig. S2), whereas the term proportional to $(\Gamma p_+/r')e^{ikr'}$ represents the wave propagating away from the aperture (increasing r'). (The complex number Γ is determined by the wave reflected at the interface at $z = -t$.) In Eq. (S2), the average pressure $\bar{p}$ and the volume velocity q can be expressed as

$$\begin{aligned}\bar{p}|_{z=-t-h} &= \frac{p_+}{\pi r_0^2}\int_0^{2\pi} d\psi' \int_0^{r_0}\left(\frac{e^{-ikr'_h}}{r'_h} + \Gamma\frac{e^{ikr'_h}}{r'_h}\right)\sigma' d\sigma' \\ &= \frac{i2p_+}{kr_0^2}\left[e^{-ikr'_h} - \Gamma e^{ikr'_h}\right]_{h+h'}^{\sqrt{(h+h')^2+r_0^2}},\end{aligned} \tag{S6}$$

and

$$\begin{aligned}q|_{z=-t-h} &= p_+\int_0^{2\pi} d\psi' \int_0^{r_0}\left[\left(1+\frac{1}{ikr'_h}\right)\frac{e^{-ikr'_h}}{r'_h} - \Gamma\left(1-\frac{1}{ikr'_h}\right)\frac{e^{ikr'_h}}{r'_h}\right]\hat{\mathbf{r}'}\cdot\hat{\mathbf{z}}\sigma' d\sigma' \\ &= \frac{i2\pi(h+h')p_+}{\rho_0 c_0 k}\left[\frac{e^{-ikr'_h} + \Gamma e^{ikr'_h}}{r'_h}\right]_{h+h'}^{\sqrt{(h+h')^2+r_0^2}},\end{aligned} \tag{S7}$$

where ψ' is the angle measured counterclockwise from the x-axis in the xy-plane. The variable r'_h represents the distance between a point on the plane $z = -t - h$ and the origin O', whereas the length σ' is defined as $\sigma' \equiv \sqrt{r'^2_h - (h+h')^2}$. By inserting Eqs. (S6) and (S7) into Eq. (S2), the acoustic impedance Z_n can be written in the following form:

$$Z_n = Z_c\sqrt{1+1/\epsilon^2} \times \frac{\exp[-ikr_0\epsilon] - \exp[-ikr_0\sqrt{1+\epsilon^2}] - \Gamma\left(\exp[ikr_0\epsilon] - \exp[ikr_0\sqrt{1+\epsilon^2}]\right)}{\exp[-ikr_0\epsilon]\sqrt{1+1/\epsilon^2} - \exp[-ikr_0\sqrt{1+\epsilon^2}] + \Gamma\left(\exp[ikr_0\epsilon]\sqrt{1+1/\epsilon^2} - \exp[ikr_0\sqrt{1+\epsilon^2}]\right)}. \tag{S8}$$

Here, ϵ and Z_c are defined by $h/(r_0 - a_0)$ and $\rho_0 c_0/\pi r_0^2$, respectively. In this equation, h' in Eqs. (S6) and (S7) is replaced with $a_0 h/(r_0 - a_0)$, using the geometric relation $a_0/h' = r_0/(h + h')$. The value of Z_n is determined by the complex coefficient Γ, which is calculated from the acoustic impedance Z_t at $z = -t$. Using Eqs. (S4) and (S5), Z_t is given by

$$Z_t \equiv \left.\frac{\bar{p}}{q}\right|_{z=-t} = \left.\frac{\int_S p dS}{\pi a_0^2} \Big/ \int_S \mathbf{u} \cdot \hat{\mathbf{z}} dS\right|_{z=-t}. \tag{S9}$$

Thus, Z_t can be related to Z_c using

$$\begin{aligned} Z_t =& \frac{Z_c\sqrt{1+1/\epsilon^2}}{\eta^2} \\ &\times \frac{\exp[-ikr_0\epsilon] - \exp[-ikr_0\sqrt{1+\epsilon^2}] - \Gamma\left(\exp[ikr_0\epsilon] - \exp[ikr_0\sqrt{1+\epsilon^2}]\right)}{\exp[-ikr_0\epsilon]\sqrt{1+1/\epsilon^2} - \exp[-ikr_0\sqrt{1+\epsilon^2}] + \Gamma\left(\exp[ikr_0\epsilon]\sqrt{1+1/\epsilon^2} - \exp[ikr_0\sqrt{1+\epsilon^2}]\right)}. \end{aligned} \tag{S10}$$

Here, η is defined by a_0/r_0. Rearranging Eq. (S10), Γ can written in the form

$$\Gamma = \frac{\left(1 - \frac{\eta^2}{\sqrt{1+1/\epsilon^2}}\frac{Z_t}{Z_c}\right)\exp[-ikr_0\eta\sqrt{1+\epsilon^2}] - \left(1 - \eta^2\frac{Z_t}{Z_c}\right)\exp[-ikr_0\eta\epsilon]}{\left(1 + \frac{\eta^2}{\sqrt{1+1/\epsilon^2}}\frac{Z_t}{Z_c}\right)\exp[ikr_0\eta\sqrt{1+\epsilon^2}] - \left(1 + \eta^2\frac{Z_t}{Z_c}\right)\exp[ikr_0\eta\epsilon]}. \tag{S11}$$

By inserting Eq. (S11) into Eq. (S8) to eliminate Γ, the acoustic impedance Z_n can be expressed as a function of the geometric variables of the horn and the impedance Z_t. This approximate model can be validated using FEM (COMSOL 5.3a). Considering the rigid conical horn waveguide configuration depicted in Fig. S2, with the dimensions as defined in the main text, we calculate Z_n for the case in which $Z_t = (1 - i)\rho_0 c_0/\pi a^2$. The mass density ρ_0 and the sound velocity c_0 of air are assumed to be 1.2 kg m^{-3} and 343 m s^{-1}, respectively. The results calculated using the analytical model are presented in Fig. S3 alongside the FEM results, showing good agreement.

2. Derivation of the impedance Z_t

The radius a of the hole in which the membrane is mounted is assumed to be much smaller than the wavelength. Using the lumped element approximation (20, 35), the impedance Z_t

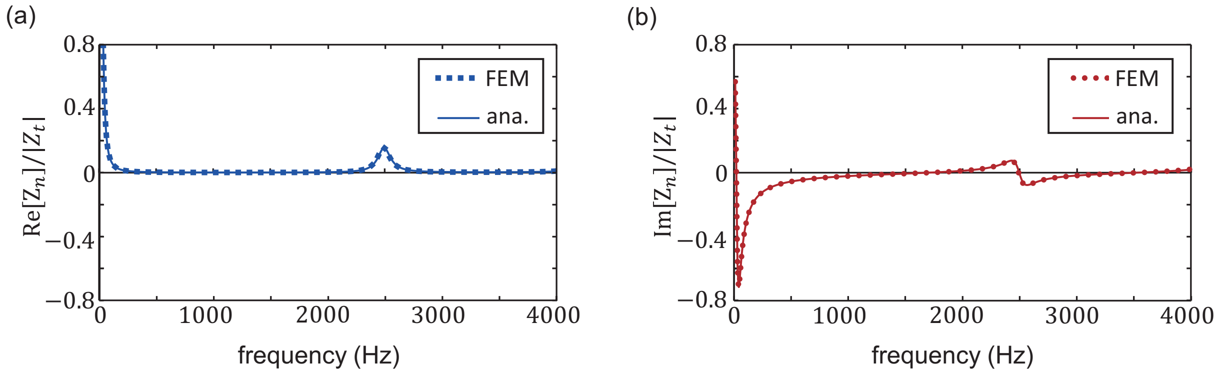

Fig. S3: (a) and (b): Real and imaginary parts of the impedance ratio $Z_n/|Z_t|$, respectively. (a) FEM: dotted blue line; analytical model: solid blue line. (b) FEM: dotted red line; analytical model: solid red line. Analytical is abbreviated in the label to "ana."

is approximately given by

$$Z_t = -i\omega\left[\frac{\rho_0(\Delta t + t)}{\pi a^2} + \frac{m_{\text{mem}}}{(\pi a^2)^2}\right] - \frac{\zeta\tau}{i\omega(\pi a^2)^2} + \frac{b_h}{(\pi a^2)^2} + Z_0, \tag{S12}$$

where m_{mem} and τ are the total mass and the pre-tension of the membrane, respectively, and b_h is a dissipative damping term associated with the hole. The dissipative term represents viscous losses associated with fluid motion inside the hole and near the hole edges, as derived analytically in Ref. (23), and also includes a contribution from membrane damping. The spring constant of the vibrating membrane is given by $\zeta\tau$, where ζ is a positive real number that depends on the shape of the vibrating membrane (at low frequencies, ζ=8π) (41). Δt is the end correction at the left side ($z = -t$) of the hole. In this equation, the impedance Z_t is determined by Z_0, which is defined as the ratio of the average pressure to the volume velocity on the surface as $z \to 0^-$. Z_0 depends on the geometry of the region $z > 0$. Its calculation is beyond the scope of the present work.

2 Experiment on a polyurethane foam sample

To verify that the experimental technique can be extended to discriminating materials of markedly different acoustic impedance, we compared the point response for two samples: a 80 mm length, 40 mm width and 6 mm thickness reticulated polyurethane foam sample, and the medium density fiberboard (MDF) wood sample (which served as the reference for 1D scanning), with air backing in each case. The imaging distance is again set to z = 2 mm for this experiment, and the same 1340 Hz frequency is used. The acoustic reflection coefficient inside the waveguide $|R|$, is measured to be a factor $\sim$ 0.9 times lower

for the foam sample than for the MDF sample, demonstrating that the zero-mass meta-atom probe can distinguish objects of different acoustic impedance. Literature values for the acoustic reflectance (i.e., corresponding to that external to the conical probe) of similar polyurethane foams for various thicknesses and backings show values of acoustic reflection coefficient differing significantly from the value of ~ 1 (expected for the MDF sample), which is consistent with our findings (42, 43).

Other Supplementary Material for this manuscript includes the following:

Movie S1 (mp4 format). Animations of the EAT probe at 1340 Hz with and without an object.
